# Diatom frustules protect DNA from ultraviolet light

**DOI:** 10.1038/s41598-018-21810-2

**Published:** 2018-03-23

**Authors:** Luis Ever Aguirre, Liangqi Ouyang, Anders Elfwing, Mikael Hedblom, Angela Wulff, Olle Inganäs

**Affiliations:** 1 Biomolecular and organic electronics, Department of Physics, Chemistry and Biology, Linköping University, 581 83 Linköping, Sweden; 2 Department of Biological and Environmental Sciences, University of Gothenburg, Göteborg, Sweden

## Abstract

The evolutionary causes for generation of nano and microstructured silica by photosynthetic algae are not yet deciphered. Diatoms are single photosynthetic algal cells populating the oceans and waters around the globe. They generate a considerable fraction (20–30%) of all oxygen from photosynthesis, and 45% of total primary production of organic material in the sea. There are more than 100,000 species of diatoms, classified by the shape of the glass cage in which they live, and which they build during algal growth. These glass structures have accumulated for the last 100 million of years, and left rich deposits of nano/microstructured silicon oxide in the form of diatomaceous earth around the globe. Here we show that reflection of ultraviolet light by nanostructured silica can protect the deoxyribonucleic acid (DNA) in the algal cells, and that this may be an evolutionary cause for the formation of glass cages.

## Introduction

Since the time of Ernst Haeckels elegant illustrations of diatoms in his *Kunstformen der Natur*, the evolutionary causes for the existence of the nanostructured glass cages, frustules, confining diatoms have been discussed^1–3^. Special proteins, the silaffins and cingulins^1,4^, extract silicates from water and build the frustules along a chitin-based scaffolding network^5–7^ in geometries specific for different species. The open structure allows material transport but with some limitations; therefore, filtering has been suggested as a reason for their existence. It has also been suggested that the frustule gives mechanical protection from predators^8^; experiments have verified that mechanical strength can be improved^9,10^.

In more recent times, it was noted that the somewhat periodic 10–100 nm patterns of holes, slits and ribs, are reminiscent of the geometries of photonic bandgap structures. The first simulation to test this hypothesis used the geometry of the centric diatom *Coscinodiscus granii* to calculate the electromagnetic properties^11^. Indeed, photonic bandgap effects were found for optical frequencies for propagation in the plane of the frustule. Experimental measurements of the photonic properties of single diatoms are reported^12–22^ and for collections of diatoms or frustules. The frustules from the genus *Coscinodiscus* act as lenses focusing light in a small volume at a distance from the frustule, and this volume is constant with changing direction of light illumination^20^. It has also been noted that the frustules may act as small spectrographs, focusing photons into specific volumes inside the diatom, possibly for absorption in chlorophyll and other chromophores.

Another possibly photonic structure is formed around algae cells in coccolithophores, with microstructures of calcium carbonate forming spherical protective shells, eventually to be used as chalk. Early calculations indicated that reflection of ultraviolet radiation (UVR, 200–400 nm) would be a property in these structures, built from another material and with a related geometry^23^.

Protection from UVR may be a reason for the evolution of frustules^2^. Experimental evidence for the harmful effect of UVR on diatom growth is found for many species and conditions^24–28^, but it is also clear that diatoms can cope with relatively high UVR intensities in natural communities^26^. The protection against UVR in diatoms include mycosporine-like amino acids absorbing from 280 to 400 nm^29^, avoidance, antioxidants and repair mechanisms^29^. A major victim of high UVR exposure is DNA, with absorption in the 200–300 nm range. Repair systems for DNA in the form of photolyases^30^ driven by blue light, are present in diatoms^31,32^. The photolyases are found throughout the living world, and have evolved into the circadian sensor for higher organisms.

The periodic and quasi-periodic patterns of the diatom frustules diffract and refract UVR. DNA is damaged by exposure to UVR. We propose that a major function of the silica frustules is to protect DNA in the algal cell from UVR. To prove this hypothesis, we have carried out experimental optical studies of diatom frustules as well as electromagnetic simulations of simplified geometry models. We have used microscopy and spectroscopy in the near and far field, to characterize the interaction of UV light between 250 to 400 nm, with the diatom frustules. Finite element simulations were used to determine the electromagnetic field distribution and Poynting vector after the interaction of UVR with frustules. In our experiments we have used four diatom species; *Navicula perminuta* Grunow (NP), *Nitzschia* sp. (N), *Coscinodiscus wailesii* Gran & Angst (CW) and *Coscinodiscus* cf. *radiatus* Ehrenberg (CR). These species develop distinctive frustule geometries. The general classification, based on the frustule geometry, divide diatoms into two groups of pennate or centric diatoms. NP and N are pennate, and CW and CR are centric.

## Results and Discussions

**Figure 1:**
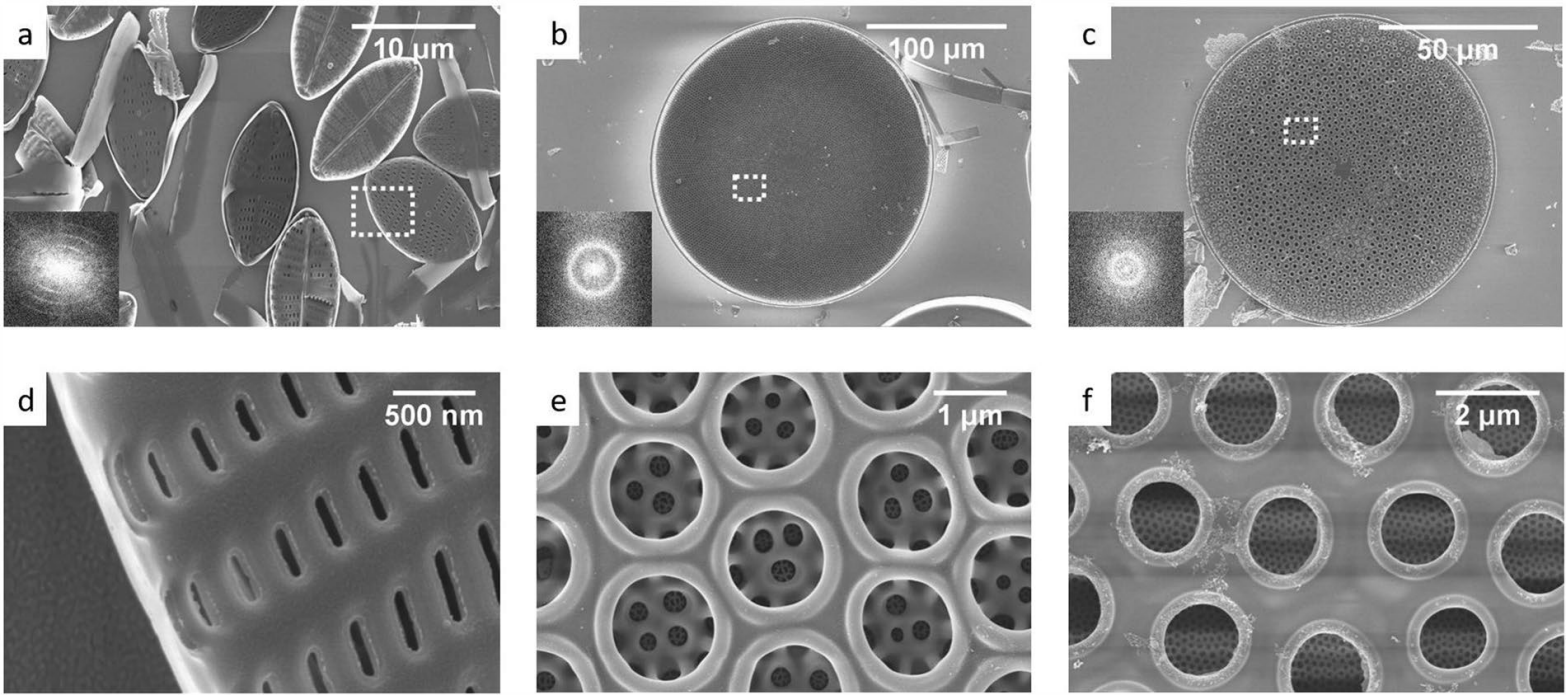
SEM images of (**a**,**d**)- *Navicula perminuta*, (**b**,**e**) *Coscinodiscus wailesii* and (**c**,**f**) *Coscinodiscus* cf. *radiatus*. Inset shows the Fourier transform for each geometry. Dashed rectangles in (**a**–**c**) correspond to the magnified areas in (**d**–**f**).

Scanning electron microscopy (SEM) images of single frustules from NP, CW and CR (Fig. 1) show a periodic arrangement of pores. The single layer structure of the NP frustules is decorated with asymmetric pores (Fig. 1d) arranged with bilateral symmetry (Fig. 1a). The size of the NP frustules is ≈10 µm apical and ≈5 µm transapical axis, respectively. The frustules of CW and CR show pores distributed with radial symmetry (at large scale) (Fig. 1b,c) with an almost perfect hexagonal arrangement (small scale) (Fig. 1e,f). Both centric diatoms exhibit more complex structures, compared with the pennate species, with a superposition of three layers with different pore sizes. The inner layer (*foramen*) include pores of diameter ≈1.4 µm placed in a hexagonal pattern. Above the *foramen* is the *cribrum*, with pores sizes ≈0.35 µm (CW) and ≈0.15 µm (CR). A third layer can be found on top of the *cribrum* containing a less well defined pattern of pores (*cribellum*); in pennate diatoms this is labeled the hymen (0.005–0.010 µm). For further insight into the terminology see^1^.

The hierarchical structure of multilevel pores in the diatom frustules can be simplified with a structure with one layer, for NP, or two layers (for CW and CR), with different pore sizes, and excluding the extremely thin hymen. The pore size and wavelengths are the most relevant parameter for scattering of incident light. The finite element method was used to solve Maxwell’s equations in the frequency domain to obtain the electromagnetic field distribution and the energy flow in the UVR range (Supplemental Information (SI)).

**Figure 2:**
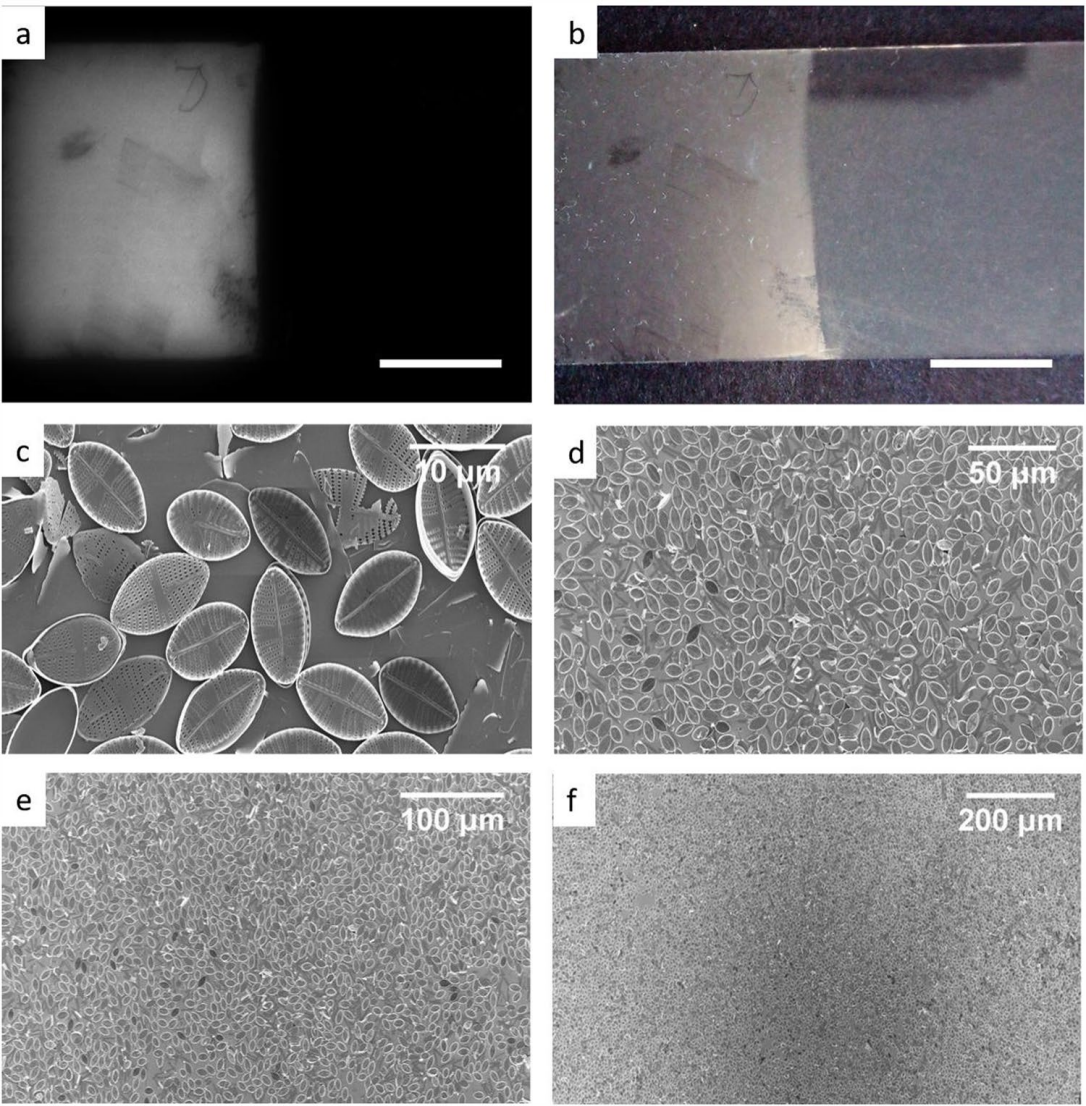
Pictures under (**a**) UVR and (**b**) visible illumination of *Navicula perminuta* monolayer on a glass slide taken in reflection mode. (**c**,**d**,**e,f**) SEM images of a NP monolayer at different magnifications. Scale bars in (**a**) and (**b**) correspond to 1 cm.

Experiments with microscopy and spectroscopy give evidence for the UVR photon flux in the near-field region close to frustules. Reflection of UVR from a monolayer of frustules (Fig. 2a) demonstrates that frustules redistribute UVR in the far field. Images of NP monolayers on a glass (SiO_2_) substrate were recorded under illumination of a UVR source (360 nm) with a UVR camera (removing all visible light) (Fig. 2a); for comparison, the reflectance image was also recorded in the visible range (Fig. 2b). SEM images of the NP monolayer verify a dense pattern (Fig. 2c–f). Measurements of NP and N monolayer transmittance and reflectance are found in SI.

The optical measurements and simulations indicate that scattering and reflection of UVR by frustules is relevant in air. With water as the internal medium of the diatoms, the difference of refractive index of the two optical media is much smaller, and the scattering and reflection effects must be smaller.

**Figure 3:**
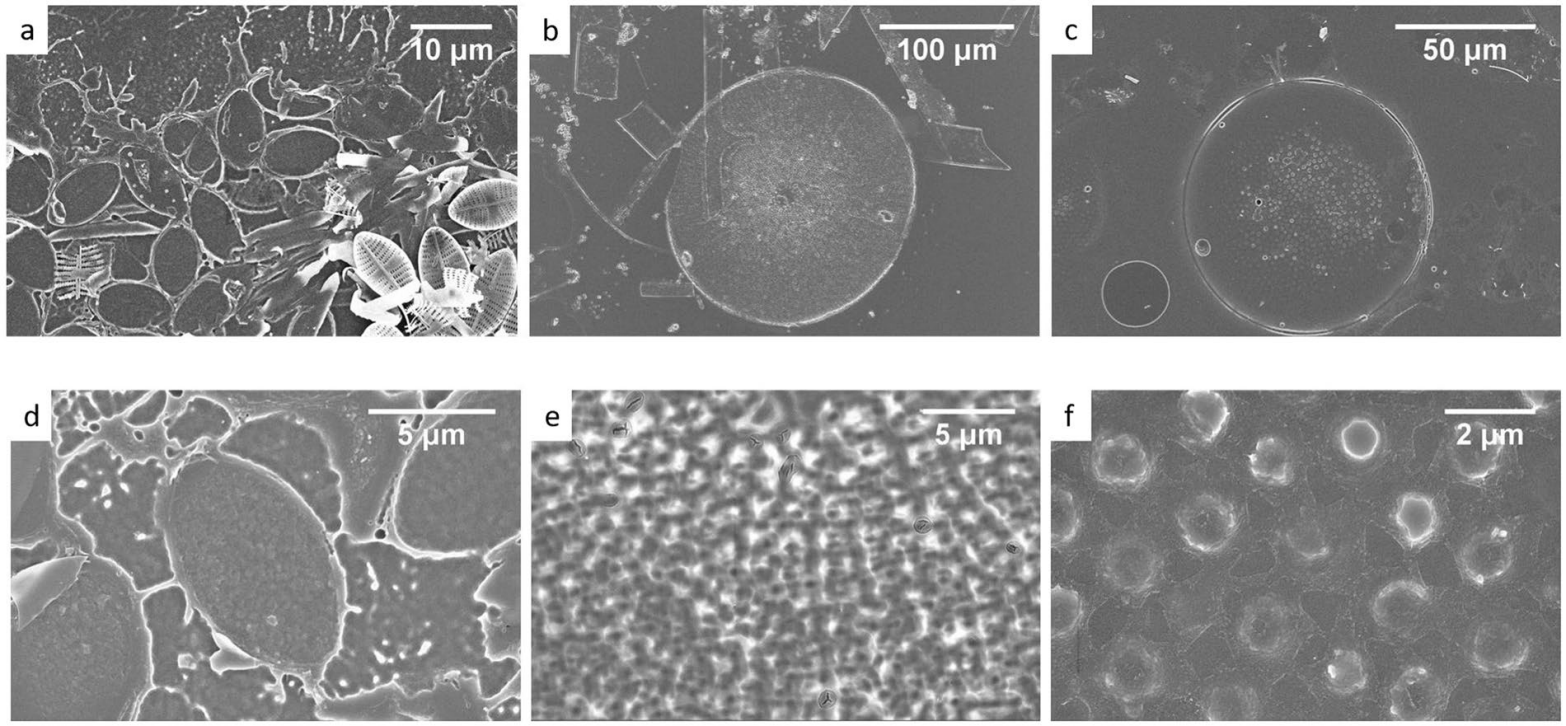
SEM images of a layer of a positive photoresist cured by a 405 nm excitation (fluorescence microscope line). Morphological detail of the area covered by a single frustule of (**a**), (**d**)- *Navicula perminuta*, (**b**), (**e**) *Coscinodiscus wailesii* and (**c**), (**f**) *Coscinodiscus* cf. *radiatus*.

The near field UVR shielding effect of frustules in air was visualized using a positive photoresist (SI). Frustules of the three species, NP, CW and CR, were dispersed on top of the photoresist layer and illuminated by a 405 nm light source for 10 s seconds, followed by development of resist. In this experiment, the amount of material removed will be proportional to the number of photons, and therefore the shielding effect due to frustules is directly visualized by the remaining photoresist. The surface morphology, after washing off the NP frustules, is imaged in SEM (Fig. 3). The strong light scattering from the frustule structures prevented the direct projection of the hole pattern on the photoresist. For frustules from centric diatoms CW (Fig. 3b,e) and CR (Fig. 3c,f), a clear difference compared with the pennate frustules was noticed. Remaining features correlated with the hole pattern, a frustule’s shadow, was observed.

The shielding effect from the diatom frustules are demonstrated when the frustules are placed between a UVR source and a UVR sensitive material. PEDOT:PSS, a widely used semitransparent organic conducting material, of relevance in organic solar cells was used. In the presence of oxygen, it is prone to UVR degradation due to overoxidation^33^, causing bleaching of the film and a decrease in conductivity. When exposing a PEDOT:PSS film to UVR through frustules spread onto the film, the frustules suppressed photobleaching of PEDOT (Fig. S4).

**Figure 4:**
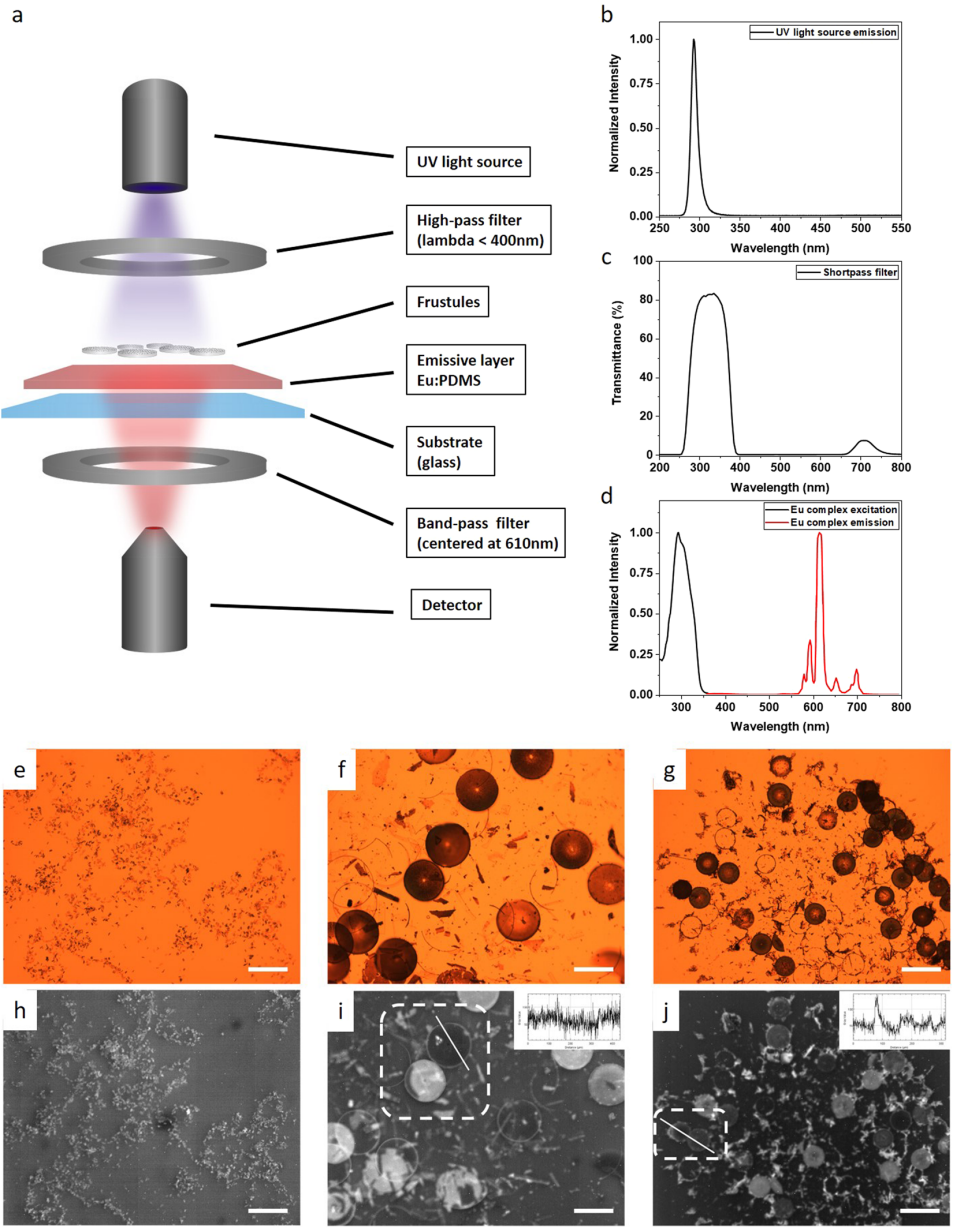
(**a**) Schematic representation of the emissive layer experimental setup, (**b**) light source spectrum, (**c**) transmittance spectrum for the short pass filter, (**d**) Eu complex excitation (black line) and emission (red line) spectra. Microscopic image of the emission of the Eu layer under different frustule geometries (**e**) and (**h**) *Navicula perminuta* (**f**) and (**i**), *Coscinodiscus wailesii*, (**g**) and (**j**), *Coscinodiscus cf*. *radiatus*. Insets in (**i**) and (**j**) correspond to the intensity values in a.u. measured over the white lines. (**e**–**g**) Images in transmission mode, (**h**–**j**) images of the emission of the Eu layer under the frustules, after monochromatic conversion and contrast digitally enhanced. Scales bars in (**e**–**j**) correspond to 200 µm.

Fluorescence microscopy was used together with a luminescent layer to visualize the energy redistribution by light scattering of the frustules. Frustules from NP, CW and CR were placed on top of an optically transparent PDMS layer stained with a phosphorescent Eu complex emitting at 600 nm. The layers were illuminated by monochromatic UVR (300 nm) in a fluorescence microscope with inverted configuration. The UVR was blocked with an integrated band pass filter. As a result, only the visible emission from the illuminated PDMS layer is detected. The emission intensity is proportional to the number of photons absorbed in the phosphorescent complex, and a uniform distribution of Eu emitters and a constant quantum yield in the PDMS layer was assumed. The image of CW frustules in transmission mode, illuminated by white light and with no filters in the optical path (Fig. 4b), is compared with the image generated by UVR in the same area (Fig. 4e), detecting only the emission of the Eu dispersed in the PDMS layer. The frustules on the film have either face-up or face-down position. With frustules in face-up position, the PDMS layer (n = 1.5 at 300 nm) and the SiO_2_ structure (n = 1.4878 at 300 nm) form close optical contact. This matching of refractive indexes gives strong light coupling, detected as a bright structure; this is because light is extracted from the guided wave in the PDMS slab beneath. This light dominates the emission from the emitter beneath the frustule. For frustules facing down, the contact area is limited to the perimeter of the frustule and light coupling is reduced. In this geometry a darker area beneath the frustule is observed (marked area), indicating that UV photons do not reach the phosphorescent compound. The inset in Fig. 4e shows the intensity profile along the white line, where inside/outside areas show lower/higher intensities, respectively.

Similar images were obtained for CR (Fig. 4g,j), where a similar interpretation can be applied. For the very small *Navicula* (Fig. 4e), frustules always outcouple light due to their small dimensions and high curvature; we cannot resolve UVR shadowing in this image.

## Conclusions

We suggest that the redistribution of UVR due to SiO_2_ frustules is an important evolutionary cause of the presence and evolution of frustules in diatoms, by decreasing the rate of UVR-induced degradation of DNA inside the cells. This will improve the energy budget for the photosynthesizing cell, as well as reduce mutations. These weak effects may be sufficient to help explain the surprising number of different diatom species with varying geometries generated by convergent evolution.

## Electronic supplementary material

Supplementary information
