# Supplementary material for: Diatom frustules protect DNA from ultraviolet light
Source: Sci Rep. 2018 Mar 23;8:5138. doi: 10.1038/s41598-018-21810-2 (PMC5865130; doi:10.1038/s41598-018-21810-2)
Supplement: Supplementary file 1 — Supplementary information [file 41598_2018_21810_MOESM1_ESM.pdf]

Title: Diatom frustules protect DNA from ultraviolet light

Authors: Luis Ever Aguirre[1], Liangqi Ouyang[1], Anders Elfwing[1], Mikael Hedblom[2], Angela Wulff[2], Olle Inganäs*[1]

Supplementary Information:

Materials and Methods

Figure S1, Pictures under (a) UVR and (b) visible illumination of *Nitzschia sp.* monolayer on a glass slide reflection mode. Scale bars correspond to 1 cm.
Figure S2, Numerical simulations of electric field and energy flow for; a and b CW, c and d CR, e and f NP along short pore axis, g and h NP along long pore axis. The frustule model is depicted at the bottom entrance for waves. The excitation wavelength is 260 nm.
Figure S3, Transmittance, reflectance and absorption spectra from NP monolayer (first row), CW and CR monolayers (second row).
Figure S4, Photo bleaching of a PEDOT:PSS layer after exposure to UVR (components between 250 and 360nm) during 24h. The light colour areas in each picture were covered during the exposure time with a- NP, b- CW and c- CR frustules. Scale bar represents 100µm.
Figure S5, SEM image of the entire frustules (a) CW, (b) CR and fine pores details (c) CW, (d) CR.

Materials and methods

Frustule preparation

The diatoms *Coscinodiscus wailesii* (CW), *Coscinodiscus* cf. *radiatus* (CR), *Nitzschia* sp. (N) and *Navicula perminuta* (NP) were cultivated separately in 0.2

µm filtered seawater (salinity 32), enriched with f/2 medium with silica. CW and CR were kept for 13 days at a temperature of ~15°C and an irradiance of ~100 µmol photons m^{-2} s^{-1} in a 16L:8D hours light cycle. NP and N were cultivated during 28 days at a temperature of ~4°C at an irradiance of ~70 µmol photons m^{-2} s^{-1} in a 22L:2D hours light cycle. Light was provided from fluorescent tubes (Osram Lumilux L36 W/865).

The diatom cultures were filtered onto 2.0 µm polycarbonate (PC) filters (Poretics Corporation) using a vacuum pump (0.2 bar), and rinsed with deionized water (dH_2O) once. Diatom cells were then resuspended in dH_2O by gentle rinsing of the filter. Cells were subsequently boiled twice in a 10% hydrogen peroxide solution at 90°C for 30 min to remove organic material from the silica shells. Diatom shells were washed with dH_2O by filtering onto PC filters. Shells were resuspended in dH_2O and frozen at -20°C and freeze dried for 48 hours into a powder.

Preparation of frustule monolayers

The frustule monolayer was created by spreading a dispersion of frustules in chloroform onto an aqueous surface. The formation of the monolayer was facilitated by a nonionic surfactant (Triton X-100, Sigma-Aldrich). The resulting monolayer was transferred to a cleaned glass slide.

SEM

Scanning electron microscopy (SEM) imaging was performed using a Zeiss Leo at 5kV acceleration voltage. Prior to imaging the samples were sputtered using a Leica EM SCD500 sputter coater with 10 Å Pt to increase contrast and enable high magnification imaging.

Numerical simulations

Finite-element method (FEM) simulations were performed using the COMSOL Multiphysics® software package solving the partial differential equations for the electromagnectic perturbation in the time domain field. In order to save computing resources, the complex structure of the frustules was reduced to a 2D representation based on the geometrical dimension extracted from the SEM images. In the case of frustules with radial symmetry, a cross section of the pores hierarchy was considered. Only two inner layers (*foramen* and *cribrum*) were included in the simulations. In order to consider the contribution of the neighboring structures, periodic conditions were applied to the boundaries along the x axis. In the case of frustules with bilateral symmetry (NP), the cross sections were taken along the apical and transapical axis. To avoid artifacts due to reflection in the boundaries perfect matching layers were applied where they were needed. Built-in refractive indexes were used for air (n=1) and SiO_2 (n=1.45).

In all cases, a plain wave incidence with a positive propagation vector parallel to the y axis and a wavelength of 260nm is considered. Based on the symmetry of the periodic arrays, the electric field source employed was polarized in the x direction and only considered the components in the x-y plane. In order to save computational resources, only a portion of the entire structure is represented. In the case of centric diatoms, only one hole of the inner plate (*foramen*) and this environment in the *cribrum* was considered. In addition, based on the hexagonal periodicity of centric diatoms, periodic boundaries along the x axis, were applied to include the contribution of the nearest neighbors. For the NP geometry, one arrangement of pores was used, considering two possible geometries; along the apical and transapical axes. In each case, the geometry used is outlined in black in Figure S2.

The electromagnetic field distribution and rate of energy flow in the near field (10µm) is shown in Figure S2a, c, e, g, after the interaction with CW, CR, NP (short axis) and NP (long axis) respectively. Figure S2b, d, f, h, correspond to the rate of energy flow for CW, CR, NP (short axis) and NP (long axis) respectively, where the white arrows represent the normalized flow direction. The electric field and rate of energy flow in the near field (10µm) after CW (Figure S2a and b) and CR (Figure S2c and d) structures reveal a stronger light scattering in comparison with the NP along short axis (Figure S2e and f) and long axis (Figure S2g and h). This effect can be related to the relation between pore size at the first interface (*cribrum*) and wavelength of the incident light (260nm) which is relevant for light scattering phenomena. Geometrical parameters were extracted from the SEM images (Fig. 1, Fig. S5), and the materials are SiO_2 and air for the surrounding medium.

Positive photoresist

The positive photoresist (Shipley MICROPOSIT S1813, sensitive to wavelengths between 350 to 450nm) was spin coated unto Si wafer at 4000 rpm (corresponding to roughly 1.5 µm thickness according to the manufacturer (http://www.microchem.com/PDFs_Dow/S1800.pdf)). The organic film was soft baked at 100°C for 5 minutes to remove solvents. A droplet of frustule containing solution was dried out for 5 minutes on the surface of the film. The film with frustules was put under a fluorescent microscope (Zeiss M200) equipped with optical filters. As illumination with red light did not affect the film it was possible to illuminate and focus on an area of interest. Switching to blue light, however, immediately started the light induced degradation process as visible in the microscope. After exposure the film for 1-5 seconds, the film was developed in 0.1 M NaOH for 1 minute where the exposed areas were washed away together with most of the frustules. The film was finally washed briefly in deionized water and blown dry with N_2.

PEDOT:PSS

PEDOT:PSS (Heraeus) was drop cast on cleaned glass and dried in air. Photodegradation was induced by exposure of the film to UVR (≈256nm plus additional components at longer wavelengths) for 24 hours. The photodegradation induced color changes.

Emissive layer

The synthesis of Eu complex was adopted from Chem. Comm. 2009, 6649 (DOI: 10.1039/b914978c). Briefly, 0.624 g (3 mmol) hexaluoroacetylacetone (Hhfac, Sigma-Aldrich) in 20 mL aqueous solution was added into 0.336 potassium tert-butoxide (KOtBu, Sigma-Aldrich). After 30 mins of stirring, 0.366 (1 mmol) $EuCl_3$ $6H_2O$ (Sigma-Aldrich) was added. The mixture was stirred under nitrogen at 60 ^oC for 1 hour then at room temperature for 3 hours. The precipitate was collected, washed with cold water and hexane and dried under vacuum for 12 hours. After drying, it was recrystallized from acetone/hexane. 0.175 mmol of the product was mixed and stirred with 0.175 mmol bis(2-(diphenylphosphino)phenyl)ether oxide (DPEPO) in 1:1 water:ethanol at 60°C for 1 hour then at room temperature for 3 hours. The final product, a white precipitate, was collected and washed with cold water and hexane and dried under vacuum. The emissive layer was made by drop casting a mixture of PDMS and the Eu complex (0.05% w/w). After curing, the Eu:PDMS film thickness was ≈ 450μm. A droplet of a solution containing frustules was dried on top of the film. The images were taken in an inverted fluorescent microscope (Zeiss M200) with an external UV light source (center wavelength at 296nm and a FWHM of 12nm). In order to suppress contribution from non-desired wavelengths, a shortpass filter was placed between the light source and the sample, only allowing transmission of wavelength below 400nm. A band pass filter in the microscope was used to pass only wavelengths around the Eu

emission (main peak at 613nm). The images were captured with an integrated camera (Zeiss AxioCam Color CCD Camera 412-312).

UV and visible reflectance of monolayers of frustules

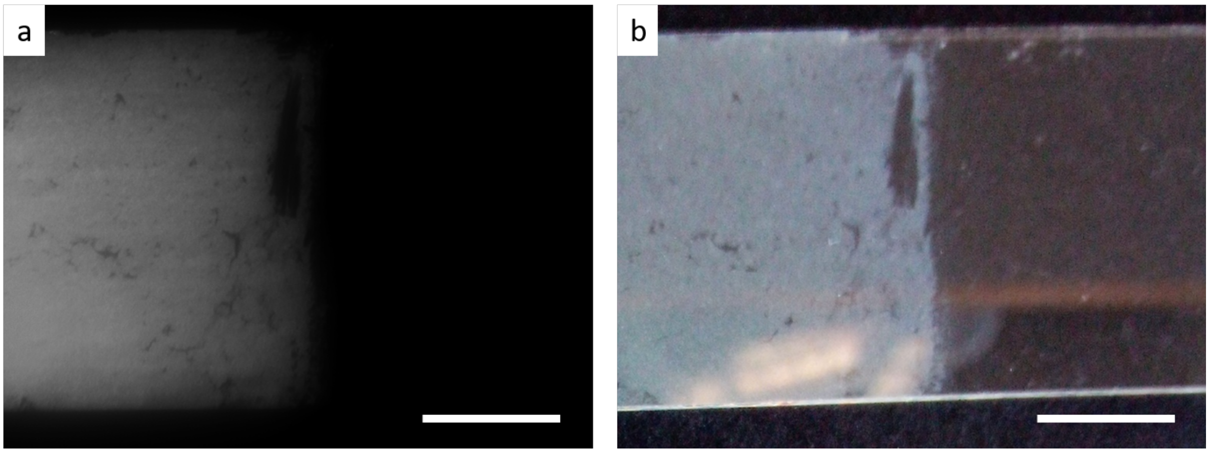

Figure S1, Pictures under (a) UVR and (b) visible illumination of *Nitzschia sp.* monolayer on a glass slide reflection mode. Scale bars correspond to 1 cm.

Electromagnetic simulations:

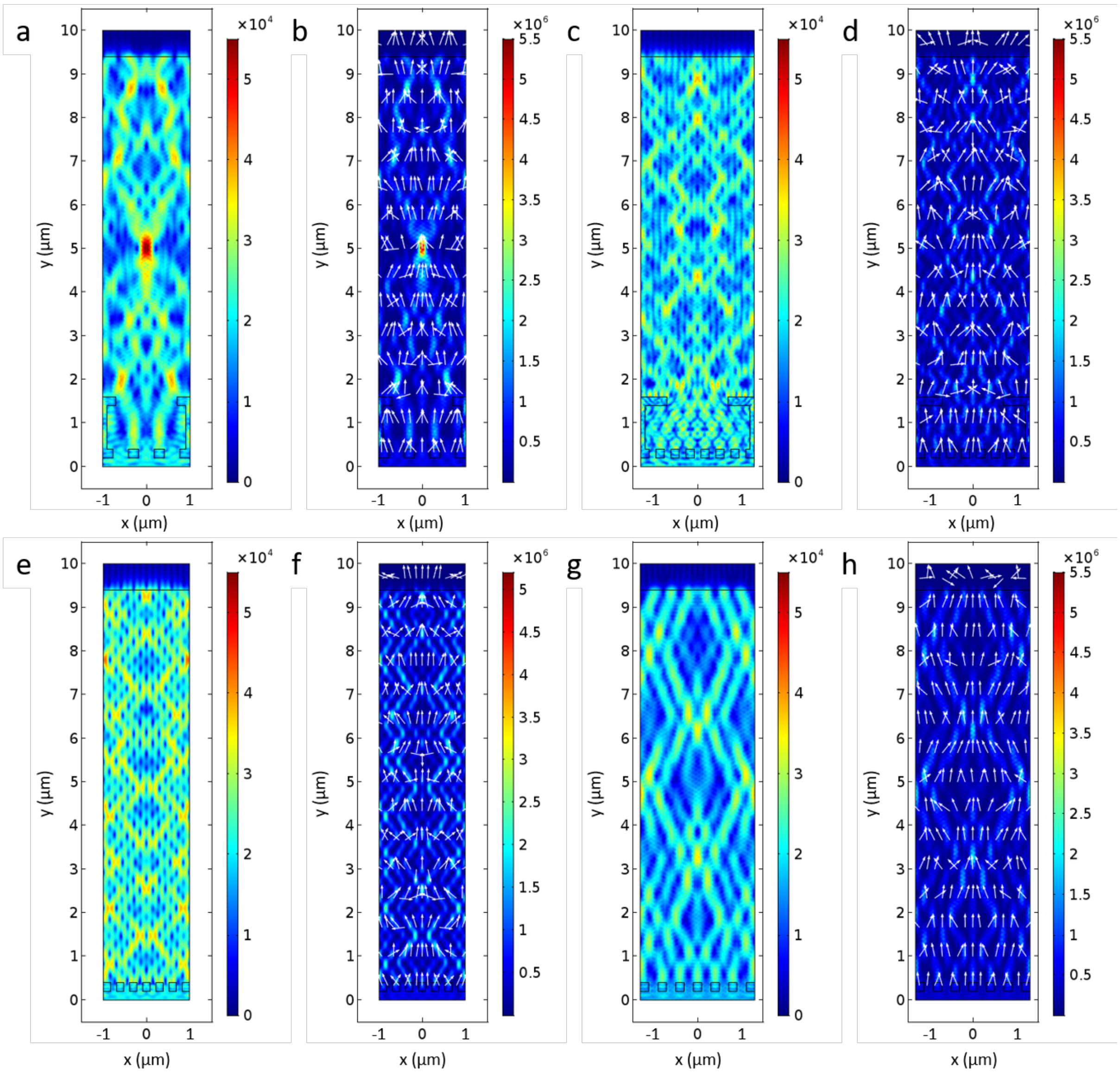

Figure S2, Numerical simulations of electric field and energy flow for; a and b CW, c and d CR, e and f NP along short pore axis, g and h NP along long pore axis. The frustule model is depicted at the bottom entrance for waves. The excitation wavelength is 260 nm.

Transmittance, reflectance and absorption spectra

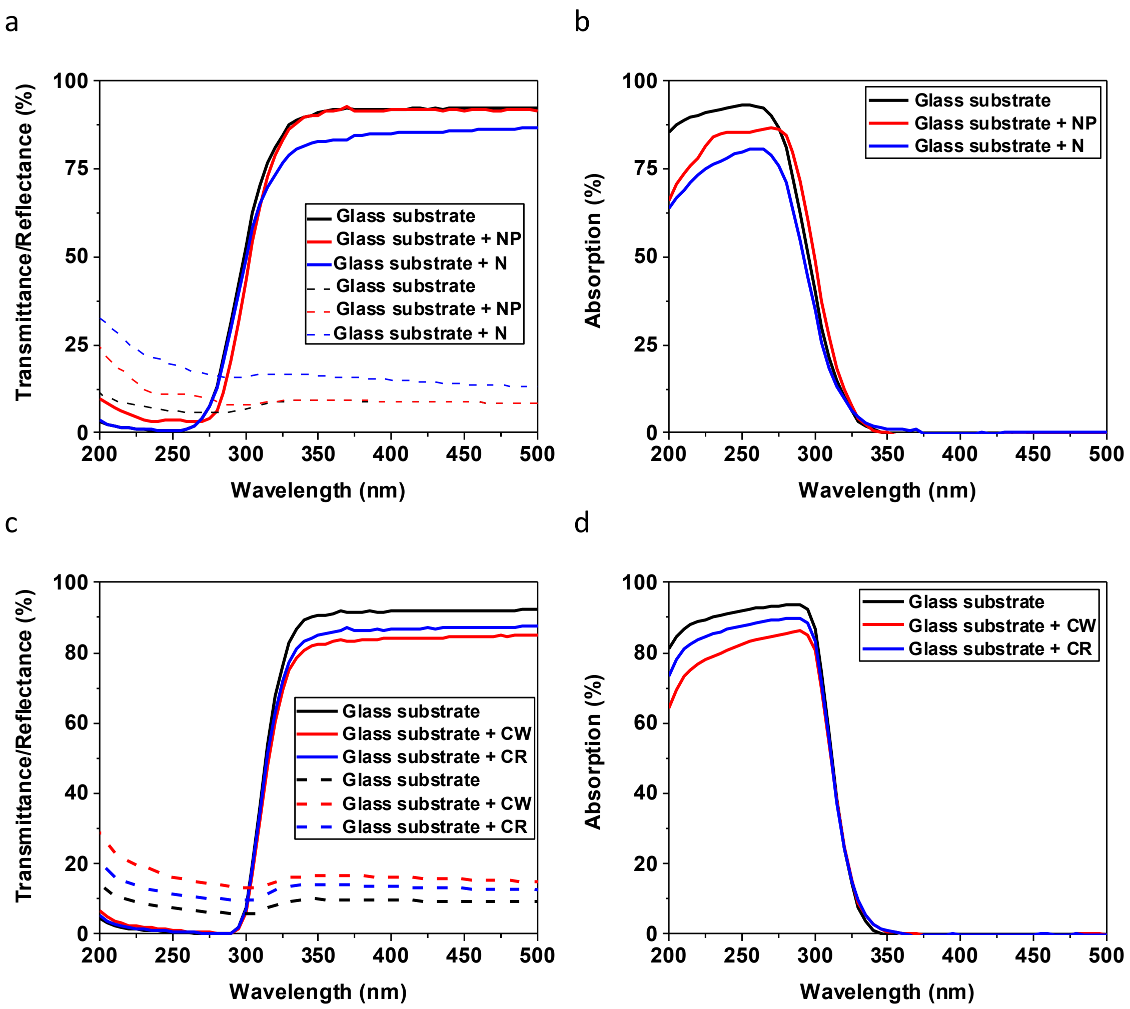

Figure S3, Transmittance, reflectance and absorption spectra from NP monolayer (first row), CW and CR monolayers (second row).

Photobleaching of a PEDOT:PSS layer

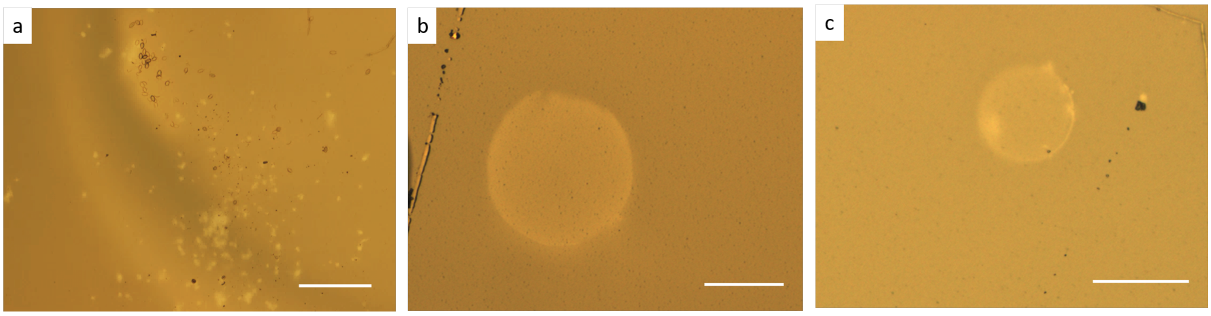

Figure S4, Photo bleaching of a PEDOT:PSS layer after exposure to UVR (components between 250 and 360nm) during 24h. The light colour areas in each picture were covered during the exposure time with a- NP, b- CW and c- CR frustules. Scale bar represents 100µm.

As shown in Figure S4, when frustules are spread onto a PEDOT film, they effectively reduce the photobleaching of PEDOT. The regions covered with show clear diatom-shaped areas on the overall bleached film, which is directly related to the wave redistribution by frustule induced UVR scattering.

SEM imaging of frustule geometries

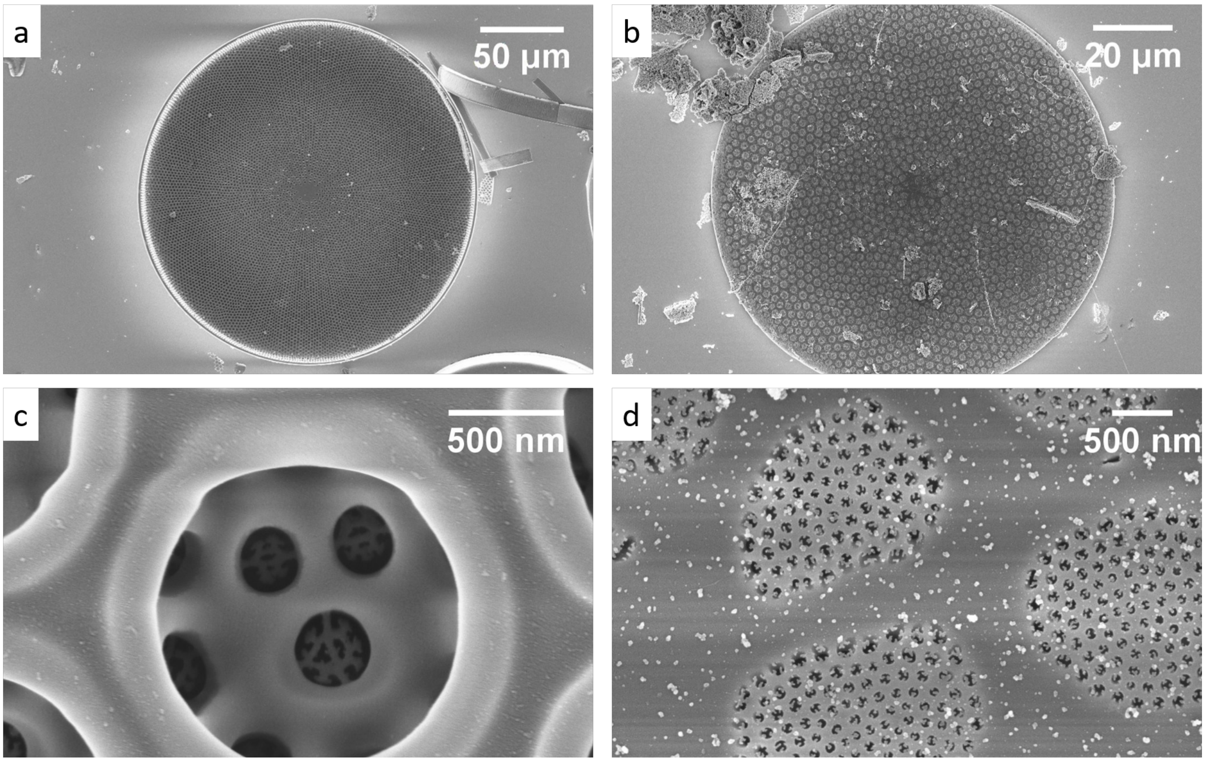

Figure S5, SEM

image of the entire frustules (a) CW, (b) CR and fine pores details (c) CW, (d) CR.
